# Composite Cryogels Based on Hydroxyapatite and Polyvinyl Alcohol and the Study of Physicochemical and Mechanical Properties

**DOI:** 10.3390/ma17020403

**Published:** 2024-01-13

**Authors:** Kseniia Shalygina, Daria Lytkina, Rustam Sadykov, Irina Kurzina

**Affiliations:** Faculty of Chemistry, Tomsk State University, 634050 Tomsk, Russia; kseniia_kolmogorova@mail.ru (K.S.); darya-lytkina@yandex.ru (D.L.); nate_river_2017@mail.ru (R.S.)

**Keywords:** cryogel, hydroxyapatite, polyvinyl alcohol, in situ synthesis

## Abstract

Nowadays, due to the increasing number of diseases and injuries related to bone tissue, there is an acute problem of creating a material that could be incorporated into the bone tissue structure and contribute to accelerated bone regeneration. Such materials can be represented by a polymeric matrix that holds the material in the bone and an inorganic component that can be incorporated into the bone structure and promote accelerated bone regeneration. Therefore, in this work we investigated polyvinyl alcohol-based composite cryogels containing an in situ deposited inorganic filler, hydroxyapatite. The freezing temperature was varied during the synthesis process. The composition of the components was determined by infrared spectroscopy and the phase composition by X-ray phase analysis, from which it was found that the main phase of the composite is hydroxyapatite and that the particle size decreases with increasing freezing temperature. The elemental composition of the surface is dominated by carbon, oxygen, phosphorus and calcium; no impurities of other elements not typical for polyvinyl alcohol/ hydroxyapatite cryogels were found. Higher mechanical properties and melting points were observed at −15 °C. Cryogenic treatment parameters did not affect cell viability; however, cell viability was above 80% in all samples.

## 1. Introduction

Repairing bone defects is an important part of traumatology and orthopedics. According to the World Health Organization, approximately 1.71 billion people in the world suffer from disorders and diseases of the musculoskeletal system [1]. In the Russian Federation, musculoskeletal diseases will account for 7.6% of total morbidity in 2020. A total of 23 million cases of injuries, poisonings and other consequences of external causes were registered. Injuries to different parts of the musculoskeletal system accounted for 16 thousand of these cases [2].

Bone tissue is a biological composite material consisting of organic and inorganic components and is characterized by a multi-layered hierarchical structure [3]. Due to its low metabolic activity, its own reparative potential is insufficient to restore large bone volumes [4]. Bone grafts, which are used to restore lost tissue, may be a suitable solution to these problems. The use of autografts is limited by the amount of bone tissue available in the body [3]. Allogeneic bone materials can cause immune rejection and the transmission of various diseases to the recipient [4]. Implantation of metallic materials can lead to osteolysis due to a significant difference in the mechanical properties of the implant and body tissues. A possible solution may be tissue engineering approaches—the creation of materials that allow full functional and structural regeneration of bone tissue.

Polymers of various origins (natural, synthetic), bioactive ceramics and calcium phosphate-based materials such as hydroxyapatite (HA) are used to restore bone defects. However, there are limitations to the use of bioceramics due to the lack of osteoinduction, brittleness and difficulty in obtaining, which is unacceptable for the replacement of large bone defects [5]. The use of polymeric materials can also be difficult because the biosorption rate of collagen and many other natural polymers exceeds the rate of bone tissue repair. Synthetic hydroxyapatite, on the other hand, is characterized by a low resorption capacity [6,7]. The development of composite materials, which include a polymer matrix with dispersed particles of calcium phosphate components, makes it possible to overcome these limitations to a large extent due to the synergistic effect of the functional properties of the components [8,9,10].

Polyvinyl alcohol (PVA) hydrogels have the necessary mechanical properties close to those of natural tissues [11,12,13], but their application in transplantology is limited by the bioresistance of the material, which prevents cell migration and attachment, which can lead to a low level of calcinosis and loose attachment of the implant to the bone, making it less suitable for integration into bone tissue [14,15,16]. The macroporous structure of the polymer matrix, which provides the similarity of the material to bone tissue facilitating the transfer processes, promoting cell migration and proliferation, can be formed by cryotropic treatment with PVA solutions. After freezing, freezing and thawing, the water–PVA system forms porous gels, cryogels. In vivo studies have shown that consolidation is more intense with cryogels [17,18,19].

Bioactive and biocompatible components, such as calcium phosphates, can be incorporated into the composition of PVA cryogel. The interaction of the components of such a system affects not only the biological activity of the composite material, but also the whole range of physical, chemical and mechanical properties. Currently, there are several approaches to obtain PVA/HA composite cryogels, including in situ deposition in PVA solutions [20,21] and dispersion of HA powder in a polymer matrix [22]. Various inorganic salts are used as precursors for the synthesis of HA. Ca(NO_3_)_2_∙4H_2_O [20] and CaCl_2_∙2H_2_O [21,23] are used as a source of Ca^2+^ ions. Phosphate ions are introduced into the system in the form of (NH_4_)_2_HPO_4_, Na_3_PO_4_ [23], NaH_2_PO_4_ [21]. NaOH ammonia buffer is often used to maintain the optimum pH [23].

The amount of foreign ions in the system, whose presence is due to the choice of such precursors, should be strictly controlled. The toxicity of nitrates is well known (the maximum permissible concentration of NO_3_^−^ is 45 mg/mL) [24]. Nitrates can form nitroso compounds with carcinogenic activity in the human body [25]. Chlorine ions provide the osmotic pressure of body fluids; their concentration belongs to a narrow range. Fluctuations in pH within a narrow range are an integral part of homeostasis. The morphology of bone tissue hydroxyapatite crystals and agglomerates is due to its hierarchical organization from the nanometer to the macroscopic scale. Synthetic hydroxyapatite obtained by various methods does not have such an organization and crystallizes in the form of agglomerates of different granulometric composition. Highly crystalline samples have low solubility and are inert in the physiological environment. Cryoprocessing parameters also vary, such as the number of freeze–thaw cycles, freezing temperature, cooling and heating rates of the system and frozen holding time.

Therefore, it is important to optimize the technology for obtaining composite PVA/HA cryogels: the selection of safe reagents, study of the influence of different parameters on the properties of the obtained materials and study of the structure and interaction mechanisms of the system components. The work is devoted to obtaining biocompatible composite materials based on polyvinyl alcohol and calcium phosphate cryogels. The aim of the work is to synthesize, study the composition, morphology, physicochemical and mechanical properties of composite materials obtained by precipitation of calcium phosphates in polyvinyl alcohol solution and the influence of parameters of cryogenic treatment.

## 2. Materials and Methods

PVA (with an average molecular weight of 104,500 g/mol) was purchased from Sigma Aldrich (Saint Louis, MO, USA), calcium oxide Sigma Aldrich (Saint Louis, MO, USA), phosphoric acid, 87 wt.% JSC REAHIM (Moscow, Russian Federation). Distilled water was used to prepare the solutions.

Composite PVA/HA cryogels were obtained by in situ deposition of HA in a PVA solution. Weighed dry reagents calcium oxide (2.23 g) and PVA (10 g) were dissolved in the calculated volume of distilled water (86 mL). The calculated amount of hydroxyapatite was 4 wt.% of the mass of the entire mixture. A ready solution of phosphoric acid (87 wt.%) was added in equimolar amounts (1.58 mL). The concentration of the solution of polyvinyl alcohol was 10 wt.%. After the solutions were drained, phosphoric acid was added to the reaction mixture. The mixture was kept under heat (T = 90 °C) in water and stirred with the help of an upper submersible bag for 14 h. At the time as the addition of phosphoric acid, the pH of the system was ≈10. By the end of the synthesis, the medium was neutral.

Ca(OH)_2_ + H_3_PO_4_ → Ca_10_(PO_4_)_6_(OH)_2_ + 18H_2_O (1)

After synthesis of the composites, the reaction mixture was brought to room temperature, followed by cryogenic treatment at −10, −15, −20 °C. The detailed data of cryogenic treatment are presented in Table 1. When a polyvinyl alcohol solution is physically cross-linked, intra- and intermolecular hydrogen bonds are formed due to the orientation of the -OH groups relative to each other [26,27]. In this regard, one of the approaches to forming a cryogel may not be repeated freezing–thawing, but slow freezing–thawing during one cycle.

In this way, three types of samples were obtained, differing in the minimum temperature of cryotreatment. For the investigations (XRD, SEM, EDX, IR), cryogels were cut in the direction perpendicular to the height of the cylindrical sample. The samples were then dried to a constant weight in a vacuum oven (T = 70 °C, p = 80 mbar).

The phase composition of the starting components and composites was determined using a MiniFlex 600 diffractometer (Rigaku, Tokio, Japan) Shimadzu XRD-6000. Survey photography of the samples was performed under monochromatic CuKα (α = 1.5418 Å) radiation in the reflection angular range of 2θ = 5–100° in 0.02° increments, with a voltage of 40 kV and a speed of 3 °/min. The phases were decoded and identified using the ICDD diffraction database (PDF-2/Release 2012 RDB).

The morphology was examined using a (AMETEK, Inc., Bervin, USA) Quanta 3D 200i two-beam scanning electron microscope at a voltage of 5 kV, with no metals applied to the surface. Particle size was calculated using the linear intercept method, which involves measuring the size of all particles using a straight line at a selected angle and then plotting a graph from the resulting data set. 

IR spectroscopy was performed on a Bruker Tensor 27 Fourier spectrometer using the ATR method in the 4500–400 cm^−1^ spectral range. Attenuated total reflection (ATR) is a type of IR spectroscopy. Its main advantage is the absence of any sample preparation procedure—the sample is placed in the cuvette compartment on the crystal surface and carefully pressed to the working surface of the crystal (in the case of solid sample analysis) or closed with a lid (in the case of solution analysis) using a clamping device with a micrometric screw, and the spectrum is recorded [28].

TG-DSC measurements were performed using a (NETZSCH, Selb, Germany) STA 449 F1 Jupiter synchronous thermal analysis instrument in the temperature range of 25–300 °C at a heating rate of 10 K/min in an argon atmosphere. Weighed, freshly prepared cryogels were placed in an aluminum crucible.

The degree of crystallinity was calculated by Formula (2):

(2) $$X_{s}=\frac{∆H_{m}}{{∆H}_{m}^{0}}\times 100\%;$$

where $∆H_{m}$ is the sample melting enthalpy; ${∆H}_{m}^{0}$ is the enthalpy of melting of completely crystallized PVA at the equilibrium melting temperature Tm, 138.6 J/g [29].

Compressive strength measurements and Young’s modulus calculations for PVA/HA specimens were performed using a setup based on the (Arduino, Scarmagno, Italy) Mega 2560 board and a strain gauge at 20% specimen deformation. The dependence of the specimen deformation on the compressive load was determined.

To assess the solubility of HA in the composite cryogels and the swelling behavior of the material, 12 mL of phosphate buffered saline (PBS, LLC PanEco, Moscow, Russia) was added to 0.500 g of the sample and kept at 37 °C for 4 weeks. Measurements of Ca^2+^ concentration (by complexometric titration in the presence of Eriochrome Black T with an ammonia buffer solution, pH = 10) and weighing were performed every 7 days. Student’s *t*-test was used to assess reliability at a significant level of *p* = 0.05.

The following equation was used to evaluate the swelling degree (Q):

(3) $$Q\left(\%\right)=\frac{\left(W_{t}-W_{d}\right)}{W_{d}}\times 100$$

where *W_t_* is the weight of the swollen samples at time *t* and *W_d_* it the initial weight of the samples. 

A cytotoxicity test using the Alamar blue indicator was used to assess the effect of the materials on the viability of immune system cells. Monocytes were isolated from the buffy coats of healthy donors at 37 °C for 6 days [30]. The buffy coats were obtained from the Blood Transfusion Department of the Northern Clinical Hospital (Seversk, Russia). The obtained monocytes were cultured at a concentration of 10^6^ cells/mL in the X-VIVO 10 medium (Lonza, Verviers, Belgium) supplemented with 1 ng/mL of M-CSF (Peprotech, Hamburg, Germany) and 10^−8^ M of dexamethasone (Sigma Aldrich, Darmstadt, Germany). Signal intensity was measured using a Tecan Infinite 200 microrider at a wavelength of 540 nm.

Institutional Review Board Statement: The study was conducted according to the guidelines of the Declaration of Helsinki and approved by the Ethics Committee of Tomsk State University (Protocol of the meeting of NR TSU Bioethics Commission, Protocol No 3 from 7 March 2022). Informed consent was obtained from all subjects involved in the study. Written informed consent has been obtained from the patient to publish this paper.

## 3. Results and Discussion

### 3.1. Determination of the Phase Composition

**Figure 1:**
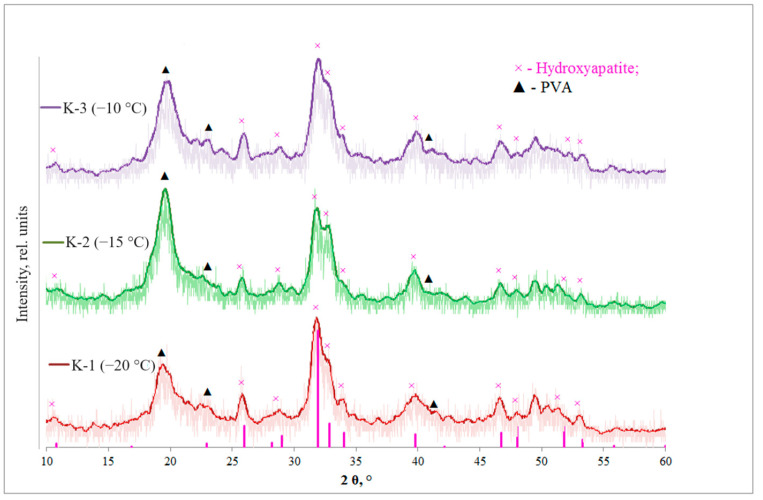
X-ray diffraction patterns of polyvinyl alcohol/ hydroxyapatite (PVA/HA) composite cryogels.

Diffraction patterns were obtained for the composites K-1, K-2 and K-3 (Figure 1).

The most intense reflections 2θ = 31.7°, 32.2°, 32.9° corresponding to HA (JCPDS N 9–432) are not resolvable using Cu Kα radiation and are represented by a peak in the range 30.62 ≤ 2θ ≤ 31.9. The most intense peaks for PVA appear around 2θ = 19.4°; 23.5°; 40.3°, corresponding to its semi-crystalline structure.

In previous studies, we have shown that the formation of the HA phase in a polyvinyl alcohol environment can be influenced by temperature and the choice of initial reagents [19]. For example, the use of salts, instead of reacting directly to neutralize the alkali with an acid at a lower temperature (50 °C), leads to the formation of monetite and brushite. Such a system is easier to control, because it is homogeneous and the evaporation of the solvent occurs less intensely, but this method does not allow hydroxyapatite to be obtained.

There is a decrease in the degree of crystallinity of HA as the freezing temperature increases. For the polymer phase, the change in the degree of crystallinity is non-linear and has an extreme character, which is characteristic of the crystallization of polymers. Probably T = −15 °C is close to the optimum values of nucleation rate and viscosity of the medium [31]. For the reflections of hydroxyapatite (211) and PVA (101), the size of crystallites values and the degree of crystallinity were calculated (Table 2).

According to the literature, during the first freeze–thaw cycle, a few small crystallites (about 3–8 nm in size) are formed, connected by swollen amorphous chains. The approximate distance between the crystallites is 30 nm [32].

The size of the crystallites of both phases does not depend on the type of cryotropic treatment. In the work of VI Lozinsky [26,33], it is shown that cryostructuring in water–PVA systems occurs at the stage of thawing.

An increase in cell parameters compared to stoichiometric hydroxyapatite is observed. This may be due to the high proportion of amorphous component. For K-2, the maximum HA cell volume is observed and for K-1 the unit cell volume is close to the values of stoichiometric HA.

Thus, the freezing temperature affects the structure of the obtained composites: the degree of crystallinity of HA decreases with an increasing freezing temperature. For PVA, the change in the degree of crystallinity is non-linear; D of both phases does not depend on the type of cryotropic treatment; the parameters of the HA unit cell change non-linearly. The cell volume is maximum for K-2 (freezing temperature −15 °C).

### 3.2. IR Spectroscopy

**Figure 2:**
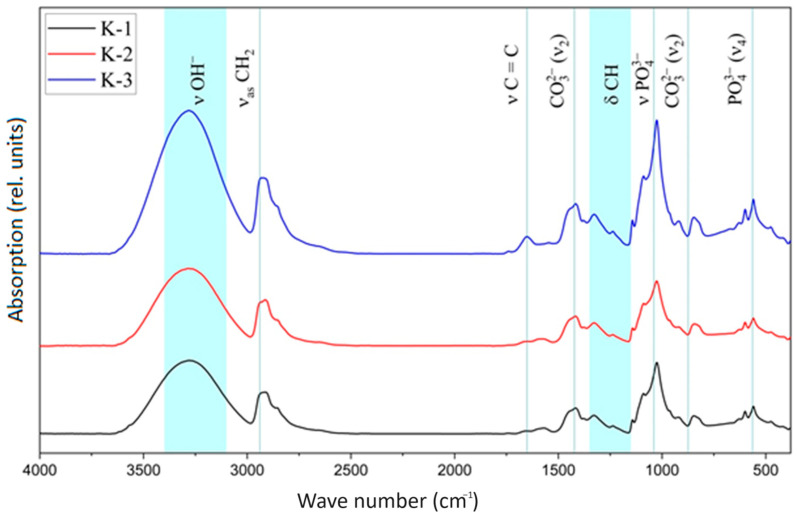
IR spectra of composite PVA/HA cryogels K-1 (−20 °C); K-2 (−15 °C); K-3 (−10 °C).

In the IR spectrum of pure PVA, there is a broad band at 3400–3100 cm^−1^ associated with the stretching vibrations of the OH groups (ν O-H, blue on Figure 2). The doublet at 2900 and 2940 cm^−1^ is associated with the CH_2_ group, while the peak with a large wavenumber belongs to asymmetric and a smaller one to symmetric stretching vibrations. The bands at 1651 and 1090 cm^−1^ belong to the C=C and C-O(H) stretching vibrations, respectively. The presence of C=C bonds may be related to intramolecular dehydration:

$$-{\left[CH(OH)-CH_{2}\right]}_{n}-\overset{H^{+}}{\to }-{\left[CH^{+}-CH_{2}\right]}_{n}-+H_{2}O\underset{-H^{+}}{\to }-{\left[CH=CH\right]}_{n}-+H_{2}O$$

The band at 1432 cm^−1^ belongs to scissor vibrations of CH_2_ groups (δas). The peak at 1710 cm^−1^ is associated with C=O and C-O bonds of acetate groups. The informative region is represented by a band at 1140 cm^−1^ associated with C-O-C stretching vibrations and torsional vibrations δCH (blue on Figure 2) [32]. Any tetrahedral ions or molecules (e.g., phosphate ions) exhibit four types of vibrational modes, such as ν1, ν2, ν3 and ν4 [34] (Figure 2).

The first sign of HA formation is a broad band with a maximum at about 1000–1100 cm^−1^ [35]. In addition, peaks at 3570 and 630 cm^−1^ are characteristic bands of stoichiometric HA [35]. The band at 962 cm^−1^ refers to non-degenerate symmetric stretching vibrations (ν1) of the P-O bond of the phosphate group [36]. The doubly degenerate ν2 mode of the phosphate group gives a faint band at 474 cm^−1^ [35]. The most intense band of the phosphate group related to the triple degenerate P-O stretching vibrations, ν3, is located at about 1094 cm^−1^. The band between 564 and 604 cm^−1^ belongs to the triple degenerate deformation mode of the P-O-P bond of the phosphate group (ν4), which occupies two positions in the crystal lattice [35]. Two peaks at these frequencies confirm the presence of two different positions of the phosphate group in the hydroxyapatite lattice. Two bands at 633 and 3546 cm^−1^ are associated with libration and stretching vibrations of the hydroxyl group in the HA crystal structure. The peak at 1635 cm^−1^ relates to the deformation vibrations of adsorbed water. During the synthesis of hydroxyapatite in an alkaline medium (pH = 10.5), atmospheric CO_3_^2−^ is absorbed and bands appear in the IR spectrum at 1422 and 875 cm^−1^ corresponding to vibrations in CO_3_^2−^. The band at 875 cm^−1^, corresponding to ν2 deformation vibrations of CO_3_^2−^, may indicate a B-type substitution. The ν3 valence mode of CO_3_^2−^ manifests itself at 1422 cm^−1^ [37]. The shape of ν3 and the absence of a band at 700 cm^−1^ related to the C-O bond indicate that calcite is associated with HA. As CO_3_^2−^ is present in bone tissue hydroxyapatite, its presence in synthesized materials may improve their biocompatibility [35].

The IR spectrum of HA contains an intense doublet at 1091 and 1039 cm^–1^ (1048 cm^–1^ [31]), corresponding to the asymmetric stretching vibrations of the phosphate group (ν3), and the band at 962 cm^–1^ belongs to its symmetric vibrations (Table 3).

The shift of the bands in the IR spectrum indicates the existence of an interaction between the mineral and gel components of the system. Characteristic bands of the carbonate ion are observed. 

### 3.3. Morphology and Elemental Composition (EDX) of Materials Surface

Surface properties determine the bioactivity of the material. Filamentous HA particles are often carcinogenic.

The morphology of bone tissue hydroxyapatite crystals and agglomerates is due to its hierarchical organization from the nano- to the macroscopic scale. Synthetic hydroxyapatite obtained by various methods does not have such an organization of crystallites in the form of agglomerates of different granulometric composition. Highly crystalline samples have low solubility.

The mechanism of bone tissue formation is its main characteristic. First, an organic fibrillar scaffold of collagen and non-collagen proteins is formed. This scaffold is a matrix that determines the spatial relationships of the deposition of hydroxyapatite crystallites on it.

The collagen scaffold is a multi-layered, hierarchically organized structure. The elements of the structure are arranged in a helical pattern at all levels. The lowest (molecular) level is a long helical molecule. At the second level, several collagen molecules are coiled into a microfibril, and so on. In total, seven levels of bone organization are considered. Hydroxyapatite crystallites, which are deposited on the collagen matrix and then fused into a single mineral monolith, repeat the entire helical hierarchical organization from the nanometer to the macroscopic level.

**Figure 3:**
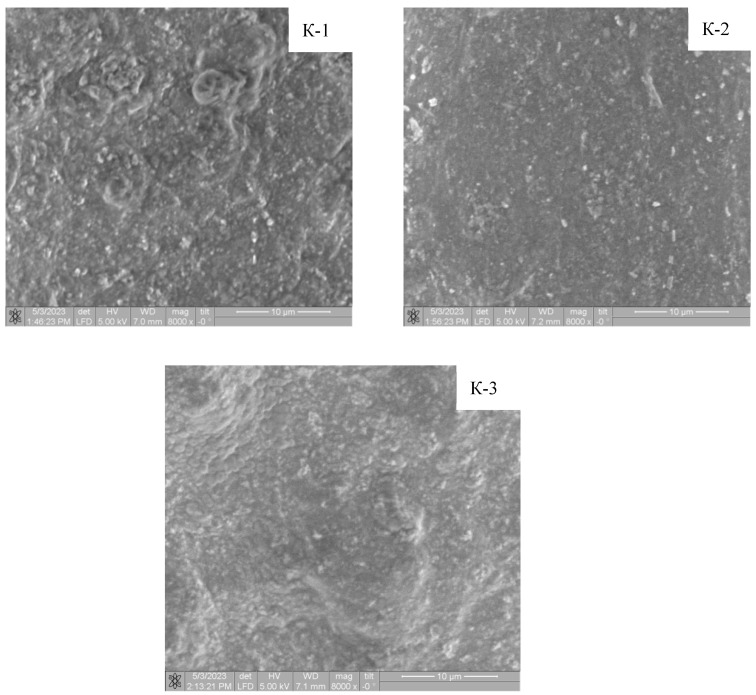
Scanning electron microscope images of PVA/HA composite cryogels.

The SEM images (Figure 3) show that the calcium phosphates crystallize in the form of agglomerates. As the freezing temperature decreases, the particle size distribution widens and becomes polymodal. Prior to cryogenic treatment, the system was not subjected to ageing and the HA phase was not finally formed. A decrease in the proportions of [33] can lead to an increase in supersaturation on the one hand and an increase in the viscosity of the medium on the other [42]. A stronger ap approximation is not possible because the polymer samples start to degrade as the stress increases.

**Figure 4:**
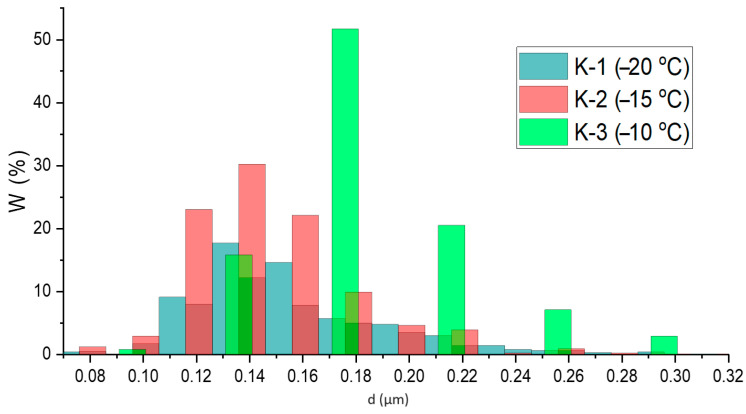
Particle size distribution histograms.

Calculation of particle size from the images (Figure 4, Table 4) showed that sample K-1 is characterized by a polymodal distribution with maxima localized in the ranges 0.101–0.111 μm; 0.121–0.131 µm; 0.161–0.181 µm. The size distribution of the particles in samples K-2 and K-3 is unimodal with maxima in the ranges 0.121–0.141 µm and 0.136–0.156 µm, respectively.

The composition of the obtained materials is dominated by carbon, oxygen, phosphorus and calcium; impurities of other elements not typical for PVA/HA cryogels were not found.

For pure hydroxyapatite Ca_10_(PO_4_)_6_(OH)_2_, the stoichiometric ratio of the elements is Ca/P = 1.67, although in natural bone tissue structures this ratio can vary within Ca/P from 1.3 to 2.1 [43]. The Ca/P ratios in the samples are significantly higher than the stoichiometric value (1.67) (Table 5). This may be due to the high proportion of amorphous component. The closest to the stoichiometric Ca/P ratio, equal to 1.87, is sample K-2.

### 3.4. Contact Angle and Surface Energy

The study of surface wetting is very important for biocompatible materials, because the biocompatibility of a material relates to the behavior of cells in contact with a surface, where the surface characteristics of the materials, such as surface topography, chemical composition or surface energy, play an important role in the adhesion process. The quality of this first phase of cell–material interaction influences and ensures good proliferation and differentiation of cells on the surface [44,45]. There are studies that suggest that increased wettability (as indicated by a low contact angle) will lead to improved biocompatibility [46,47].

The wetting angle (θ, °) was determined by the sessile drop method for all PVA/HA composite cryogels and starting components. Water and glycerol were used as wetting liquids. All materials and components are hydrophilic in nature as θ water < 90°.

Despite the different chemical nature of the components of the system, we found that the total surface energy (72–75 mJ) and the ratio of its polar (58–56 mJ) and disperse (9–15 mJ) components are within narrow limits and practically do not change depending on the way the composites are obtained (Table 6 and Table 7). Similar values of wettability and surface energy suggest that with similar chemical compositions of the surfaces of the materials, cells will demonstrate similar viability in the presence of materials.

### 3.5. Thermal Analysis

**Figure 5:**
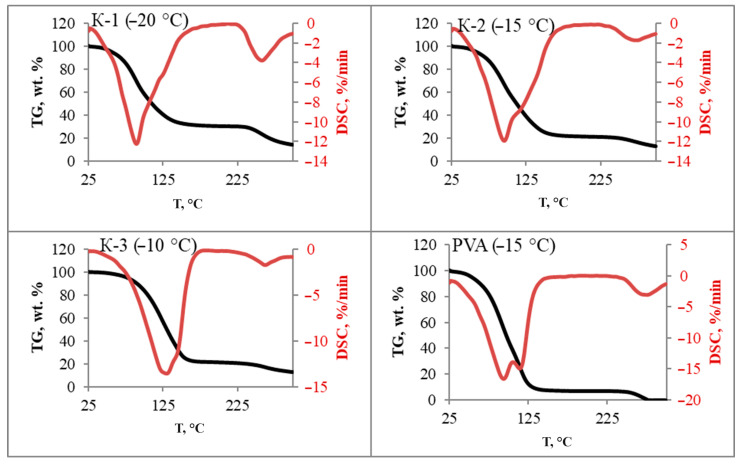
TG and DSC curves for PVA/HA composite cryogels.

The thermograms (Figure 5) show a two-stage weight loss. Dehydration occurs over a wide temperature range and is accompanied by endo effects corresponding to the destruction of the spatial structure of the gel. A further increase in temperature causes thermal degradation of the polymer.

On the DSC thermograms of PVA (95.8; 116.3 °C) and K-3 (129.6; 140.3 °C), two peaks are clearly distinguishable, while for K-2 a peak at 95.3 °C and a bend at 119.1 °C are determined. The curve for K-1 has a peak at 89.1 °C and is then monotonic up to the melting point of the PVA crystallites.

The peak at 85 °C is called α-relaxation and represents the glass transition temperature of PVA. In the presence of water and other solvents, the glass transition temperature decreases significantly [29]. The β-relaxation at 143 °C corresponds to the relaxation in crystalline domains. The third relaxation occurs at temperatures between 200 and 260 °C due to melting of the crystallites. The end effect at 90 °C indicates the secondary formation of crystallites. The sol-gel transition of PVA during the formation of physically crosslinked hydrogels occurs at 55–70 °C [48,49].

The sol-gel transition temperature varies non-linearly and is maximum for K-3, which may indicate the strongest intermolecular interactions. The end effects corresponding to the secondary formation of PVA crystallites and melting of the polymer decrease for composites of relatively pure PVA. It is likely that the presence of HA prevents PVA–PVA intermolecular interactions. However, the melting temperature of the polymer in the K-2 composite is comparable to that of pure PVA cryogel. For the same sample, the maximum degree of crystallinity is observed in the series of composite cryogels.

### 3.6. Study of Mechanical Properties

**Figure 6:**
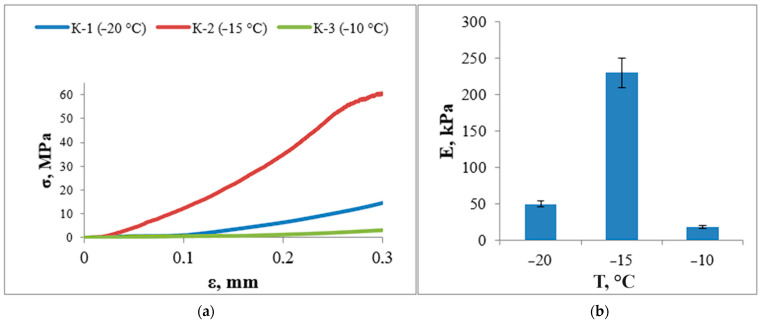
Mechanical properties of PVA/HA cryogels. (**a**) Stress–strain curves; (**b**) the effect of freezing temperature on Young’s modulus.

In order to determine the effect of the cryotropic treatment temperature on the modulus, materials were obtained at different initial freezing temperatures; as the initial temperature is increased from −20 °C to −10 °C, the modulus changes non-linearly (Figure 6).

The maximum value of Young’s modulus is observed for materials obtained at −15 °C. Apparently, the high degree of crystallinity of PVA in sample K-2 has a direct effect on its mechanical properties. According to other studies [49], for cryogels obtained by repeated freezing and thawing at −15 °C from 10 wt.%, the selected mode allows such properties to be obtained in one cycle. The increase in Young’s modulus is directly related to the degree of crystallinity of the sample, as there is an increase in intermolecular interactions as a result of an increase in the content of ordered crystalline regions. Therefore, sample K-2, which shows the highest crystallinity of PVA, has the highest elastic modulus value; this, coupled with such a parameter as the degree of swelling, may indicate that in the sample with high mechanical strength the formation of the largest number of hydrogen bonds is observed. This tells us that the temperature regime for the K-2 sample (with a maximum freezing temperature of up to −15 °C) is the most preferable.

### 3.7. Solubility and Swelling

**Figure 7:**
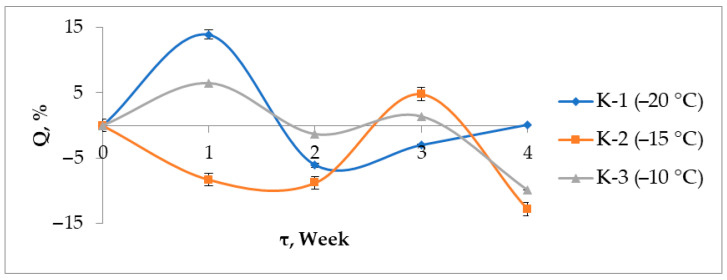
Swelling degree of PVA/HA cryogels.

The degree of swelling (Q) of the cryogels in PBS varies non-linearly, with maxima occurring in the first and third weeks (Figure 7). The degree of swelling of the gel can indirectly estimate the number of hydrogen bonds during physical cross-linking of polyvinyl alcohol. The more the gel swells, the more space there is in the spatial network of the gel for the diffusion of solvent into it. This statement correlates with the degree of crystallinity of PVA (Table 2), because for sample K-2 (obtained at a minimum temperature of −15 °C), the greatest crystallinity is observed—the greatest ordering of the segments of macromolecules, which, together with the value of the elastic modulus, indicates a greater number of hydrogen bonds in the spatial network of this sample. Therefore, we do not observe swelling for this sample in the first week.

**Figure 8:**
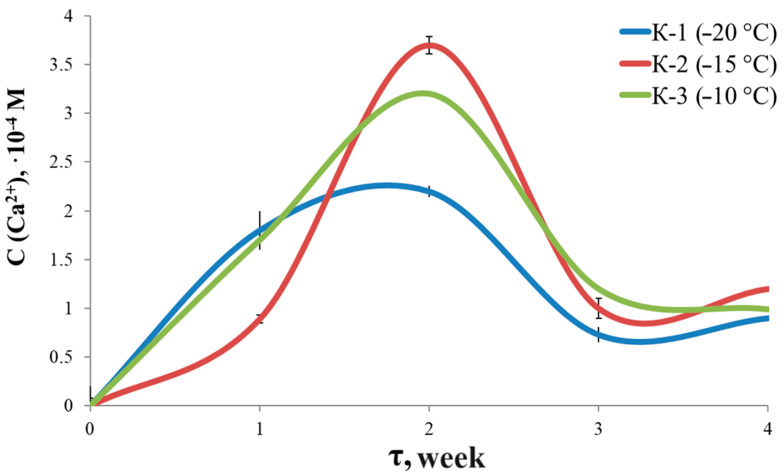
Ca^2+^ concentration curve.

This can be explained by the ageing processes of the gel as well as changes in the concentration of Ca^2+^ ions. First, the gels swell, accompanied by the release of Ca^2+^ ions. By the third week, degradation of the material leads to a significant change in structure and syneresis. The stability of the materials for 1–3 weeks varies in the series: K-1 ≈ K-3 < K-2. By the fourth week, the concentration of calcium ions becomes comparable for all samples. The maxima in the Ca^2+^ concentration curves (Figure 8) may be related to the recrystallization processes, since HA is the most stable phase under physiological conditions:

Amorphous Calcium Phosphate → HA.

### 3.8. Cell Viability

**Figure 9:**
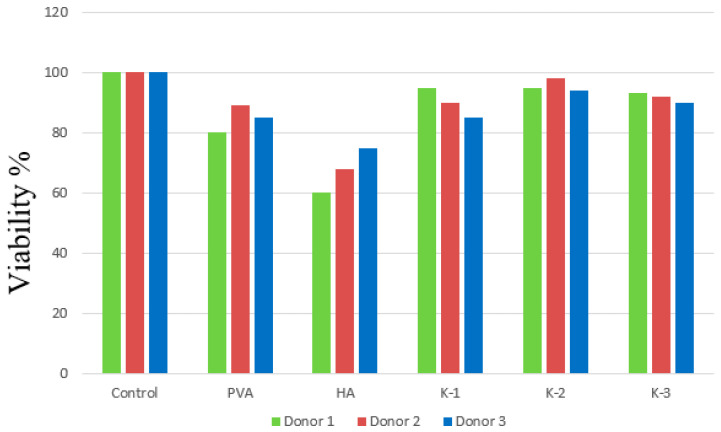
Study of the viability of macrophages in the presence of composite materials K-1, K-2 and K3, pure hydroxyapatite HA and pure PVA on three donors.

A study of the viability of monocytes in the presence of materials was carried out in the presence of composite materials, as well as individual components of pure hydroxyapatite and PVA cryogel (Figure 9). Monocytes were isolated from human blood; then, the samples were incubated at 37 °C for 6 days in the presence of materials. After incubation, Alamar Blue was added to the samples and incubated for an additional three hours. Analysis of macrophage viability showed that in the presence of pure HA, approximately 60–80% of cells survived, and in the presence of pure PVA and composites the viability was comparable to the control. The results of these studies Indicate that the material does not interfere with cell survival, suggesting that the cytotoxicity of the materials is low. Other studies [19] have shown that when a composite is prepared by mechanically mixing the finished HA powder with a PVA solution, the cytotoxicity of the material increases with increasing mass fraction of HA in the material. However, it is worth noting that these composites have a significantly higher amount of HA in the material (50–99 wt.%), which makes the contribution of HA higher. Based on the study of other composite materials [50], e.g., hydroxyapatite—a copolymer of lactide and glycolide (with an HA content in the final composites of 95, 93, 83 wt.%) using the same technique—we know that the concentration of HA is not the determining factor in the viability of macrophages. The way in which the surface of the HA particles is coated with the polymer has a significant influence. Studies of pure hydroxyapatite obtained from salts and neutralization reactions [51] also show lower macrophage viability compared to HA-PVA composites; in this case, the content of HA and PVA is 10 wt.%, which is a closer value to the present studies. This shows that it is not just the concentration of the components that contributes to the level of cell viability in the presence of materials. The production method and the starting components also make a significant contribution, making it possible to obtain materials with different surface and structural properties.

The assumption that similar values of wettability and surface energy correlate with cell viability was confirmed. However, we can observe that the viability value of macrophages in the presence of hydroxyapatite is slightly reduced compared to other materials. Perhaps this is due to the fact that despite the almost identical values of the total surface energy (Table 7), the polar component of the surface energy (σ^P^) of hydroxyapatite is higher than that of other materials, because on its surface there are practically no non-polar groups that would make a greater contribution to the dispersion component of the surface energy (σ^D^).

## 4. Conclusions

Composite hydroxyapatite–polyvinyl alcohol cryogels have been obtained by in situ synthesis. The main inorganic phase of the obtained materials is hydroxyapatite Ca_10_(PO_4_)_6_(OH)_2_. The influence of the polymer matrix is expressed in the shift of the reflections of the crystalline phase of the antisymmetry of the change in the degree of crystallinity of the polymer and mineral phases. The size of HA crystallites is weakly dependent on the type of cryotropic treatment and is apparently determined by the influence of the polymer matrix. The size of PVA crystallites decreases with a decreasing freezing temperature. An increase in cell parameters relative to stoichiometric hydroxyapatite is observed. HA is formed in the form of agglomerates of crystals with a size of 0.14–0.17 µm. The size of HA particles decreases with an increasing freezing temperature and their distribution becomes narrower. The elemental composition of the surface is dominated by carbon, oxygen, phosphorus and calcium; impurities of other elements not characteristic of PVA/HA cryogels were not found. The Ca/P ratios in the samples are significantly higher than the stoichiometric value (1.67). This may be due to the high proportion of amorphous component. The closest to the stoichiometric Ca/P ratio, equal to 1.87, is sample K-2. In addition, sample K-2 has the highest melting point of PVA and the highest mechanical properties of all the materials, which allows us to conclude that the thermal treatment mode chosen for it makes it possible to improve the interaction between the components of the system and improve the properties of the material. The concentration of calcium ions and the mass of the samples in the modelling medium change in a non-linear way. The dependence has an extreme character (maximum for a two-week exposure). The maximum calcium release is characteristic of sample K-2. The non-linear change in calcium ion concentration over time may indicate the processes of recrystallization and the reversible sorption of ions by polymer chains. The change in the mass of gels may be caused by the processes of their ageing and shrinkage, which indicates the non-equilibrium of the system under physiological conditions, a possible influence of the modelling medium (solvent exchange). The described technique reduces the number of steps compared to the existing methods of PVA-HA cryogels synthesis. It can also be used to create customized bone tissue implants. The viability study of the composite materials on three donors showed high cell viability in the presence of the materials. The cryogenic treatment mode has little effect on the viability of monocytes, but these materials can be considered biocompatible because the cells show high viability in the presence of the materials, which will allow the use of such material to fill bone defects. 

## Figures and Tables

**Table 1:** Temperature mode of cryotropic treatment.

Sample	K-1				K-2			K-3				
T, °C	−20	−7	+7	+20	−15	−7	+7	+20	−10	−7	+7	+20
τ, min	240	15	700	35	240	15	700	35	240	15	700	35
Cooling rate, °C/min	0.167				0.146			0.125				

**Table 2:** Crystallographic characteristics of composite materials.

Characteristics		Samples		
		K-1	K-2	K-3
Xs HA (211), %		22	16	9
Xs PVA (101), %		18	42	23
D (PVA) (101), nm		6	6	6
D (HA) (211), nm		10	10	12
HA crystal lattice parameters	a = b, Å	9.43	9.51	9.45
	c, Å	6.89	6.98	6.90
	V hex, Å^3^	77.01	78.32	77.34

Xs is the degree of crystallinity; D is the size of crystallites; V hex is the volume of elementary cells.

**Table 3:** Absorption bands for PVA/HA composite cryogels [31,32,33,34,35,36,37,38,39,40,41].

Vibrations	Wavenumber (WN), cm^−1^	K-1 WN, cm^−1^	K-2 WN, cm^−1^	K-3 WN, cm^−1^
ν, stretching vibrations, OH- (strong)	3400–3100	3271	3285	3281
asymmetric stretching vibrations, CH_2_	2940	2908	2914	2920
symmetrical stretching vibrations, CH_2_	2900	2854	2854	2856
stretching vibrations, C=C	1651	1655	1647	1653
Stretching vibrations, -C-O-H	1560	1572	1582	1545
scissor oscillations, CH_2_	1432	1416	1418	1416
stretching vibrations, C-O-C	1150–1060	1142	1142	1142
torsional vibrations, δCH	1150−1350	1329	1327	1327
stretching vibrations C-C bonds between the carbon of CH_2_ groups and carbon atoms related to unsaturated bonds	914	920	920	920
ν1 symmetrical stretching vibrations, PO_4_^3−^ (weak)	962	964	964	-
ν3 asymmetric stretching vibrations, PO_4_^3−^	1094	1090	1088	1090
ν3 asymmetric stretching vibrations, PO_4_^3−^	1039	1024	1026	1026
ν4 stretching vibrations, PO_4_^3−^ (strong)	564, 604	557, 598	559, 600	559
librational vibrations, OH- (middle)	633	627	627	629
stretching vibrations, OH- (weak)	3546	3271	3285	3281
ν2 deformation vibrations, OH- (weak)	1635	1655	1647	1653
ν2 deformation vibrations, CO_3_^2−^	875	847	843	847
ν3 stretching vibrations CO_3_^2−^	1422	1416	1418	1416

**Table 4:** Average particle diameter.

Sample	D, µm	σ^2^ (D, μm)
K-1 (−20 °C)	0.15	0.04
K-2 (−15 °C)	0.14	0.04
K-3 (−10 °C)	0.17	0.07

**Table 5:** Ca and P content in materials and Ca/P ratio.

Sample	Ca, At %	P, At %	Ca/P
K-1	5.32	2.73	1.95
K-2	5.60	3.00	1.87
K-3	4.63	2.29	2.02

**Table 6:** Contact angle θ for components and composite cryogels.

Sample	HA	PVA	K-1	K-2	K-3
θ water, °	9.6	10.2	9	10.2	9.3
θ glycerol, °	25.5	15.3	18.9	23.4	10.9

**Table 7:** Surface energy of composite PVA/HA cryogels and components.

Sample	σ^D^, mJ/m^2^	σ^P^, mJ/m^2^	σ, mJ/m^2^
HA	9.28	66.10	75.38
PVA	14.34	58.33	72.67
K-1	12.52	61.13	73.65
K-2	10.53	63.88	74.41
K-3	15.71	56.71	72.49

σ^D^—dispersion component of the surface energy, σ^P^—polar component of the surface energy.

## Data Availability

Data are contained within the article.
